# The Impact of Ambient Air Pollution on Daily Hospital Visits for Various Respiratory Diseases and the Relevant Medical Expenditures in Shanghai, China

**DOI:** 10.3390/ijerph15030425

**Published:** 2018-02-28

**Authors:** Hao Zhang, Yue Niu, Yili Yao, Renjie Chen, Xianghong Zhou, Haidong Kan

**Affiliations:** 1 Department of Public Administration, School of Economics and Management, Tongji University, Tongji Building A, 1500 Siping Road, Shanghai 200092, China; zhanghao@smhb.gov.cn (H.Z.); yaoyili@sina.com (Y.Y.); 2 Department of Environmental Health, School of Public Health, Key Lab of Public Health Safety of the Ministry of Education, Fudan University, P.O. Box 249, 130 Dong-An Road, Shanghai 200032, China; 17111020012@fudan.edu.cn (Y.N.); haidongkan@gmail.com (H.K.); 3 Shanghai Key Laboratory of Meteorology and Health, Shanghai 200030, China

**Keywords:** air pollution, respiratory diseases, emergency-room visits, outpatient visits, expenditures

## Abstract

The evidence concerning the acute effects of ambient air pollution on various respiratory diseases was limited in China, and the attributable medical expenditures were largely unknown. From 2013 to 2015, we collected data on the daily visits to the emergency- and outpatient-department for five main respiratory diseases and their medical expenditures in Shanghai, China. We used the overdispersed generalized additive model together with distributed lag models to fit the associations of criteria air pollutants with hospital visits, and used the linear models to fit the associations with medical expenditures. Generally, we observed significant increments in emergency visits (8.81–17.26%) and corresponding expenditures (0.33–25.81%) for pediatric respiratory diseases, upper respiratory infection (URI), and chronic obstructive pulmonary disease (COPD) for an interquartile range increase of air pollutant concentrations over four lag days. As a comparison, there were significant but smaller increments in outpatient visits (1.36–4.52%) and expenditures (1.38–3.18%) for pediatric respiratory diseases and upper respiratory infection (URI). No meaningful changes were observed for asthma and lower respiratory infection. Our study suggested that short-term exposure to outdoor air pollution may induce the occurrences or exacerbation of pediatric respiratory diseases, URI, and COPD, leading to considerable medical expenditures upon the patients.

## 1. Introduction

A number of epidemiological studies have documented that short-term exposure to ambient air pollution is associated with increased respiratory mortality and hospitalizations, especially in developed countries. In Mainland China, the largest developing country, daily mortality has been associated with particulate and gaseous ambient air pollution [1,2]. However, data on the association between air pollution and morbidity are quite scarce in the country [3]. In these limited morbidity studies, inhalable particulate matter (PM_10_, with an aerodynamic diameter less than or equal to 10 μm), sulphur dioxide (SO_2_), and nitrogen dioxide (NO_2_) were most frequently examined; however, studies have rarely focused on fine particulate matter (PM_2.5_, with an aerodynamic diameter less than or equal to 2.5 μm), ozone (O_3_), and carbon monoxide (CO), which may be more detrimental to respiratory health [3,4]. Furthermore, most of the existing morbidity studies have evaluated total hospitalizations, outpatient visits, and emergency visits and their main categorizations (cardiovascular and respiratory, etc.), leading to difficulties in fully understanding the cause-specific respiratory response to ambient air pollutants [4,5,6].

In another important aspect, the monetization of health loss associated with air pollution is becoming a critical component in policy making. Previous methods to value health costs associated with air pollution mainly include the human capital approach and willingness-to-pay method [7,8]. However, the two methods can not directly measure the true cost of disease. Up to date, few studies have linked air pollution and the direct medical cost for specific diseases.

Therefore, we conducted a systematic analysis on the associations between various air pollutants and hospitalizations of various specific respiratory diseases, as well as the associations between air pollutants and medical expenditure for these diseases in Shanghai, the largest city in China.

## 2. Materials and Methods 

### 2.1. Data Collection

The daily number of outpatient- and emergency-room visits for respiratory diseases from 1 January 2013 to 31 December 2015, was obtained from Xin Hua Hospital (affiliated to Shanghai Jiao Tong University). Located in the central urban areas of Shanghai, Xin Hua Hospital is one of the largest hospitals in Shanghai, receiving approximately 3.5 million emergency and outpatient visits per year. It is a general hospital and has a large department of pediatrics. In the emergency and outpatient departments, the physicians must enter medical record data (complaints, diagnosis, etc.) for each patient into the Hospital Information System. The medical expenditure was finally added into this system once the patient left the hospital. Further, we excluded those patients not living in Shanghai to minimize the measurement error of air pollution exposure. According to the primary diagnosis, we counted the patients diagnosed with respiratory diseases, as well as five independent categories including pediatric respiratory diseases (PRD), upper respiratory infection (URI), lower respiratory infection (LRI), asthma, and chronic obstructive pulmonary disease (COPD). We also extracted the daily medical expenditure caused by each of the above diseases. Data were analyzed at the aggregate level and no individual records/information for patients were used, so our study had a waiver of informed consent.

Daily 24-h air pollution concentrations, including PM_2.5_, SO_2_, NO_2_, CO, and O_3_, were obtained from nine state-owned air quality monitoring stations in Shanghai. According to Chinese government rules, the location of state-owned stations should not be in the direct vicinity of traffic or industrial sources. To calculate the 24-h mean concentrations of PM_2.5_, SO_2_, NO_2_, and CO, at least 75% of the 1-h values must have been available on that particular day. Daily average O_3_ is calculated by using the hourly measurement in a maximum of eight hours (10 a.m. to 6 p.m.). We calculated the averages of all valid measurements from the nine stations before entering statistical analyses. 

To allow for adjustment for the potential confounding effects of weather conditions, daily 24-h mean temperature and relative humidity were obtained from the Shanghai Meteorological Bureau.

The Institutional Review Board at the School of Public Health, Fudan University, approved the study protocol (No. 2014-07-0523) with a waiver of informed consent because all data were analyzed at an aggregate level and no participants were contacted.

### 2.2. Statistical Analyses

In environmental epidemiology, the time-series approach is widely used to investigate short-term associations between air pollution and adverse health outcomes [1,9]. This approach has the advantage of automatically controlling time-invariant confounders (such as age, sex, smoking, drinking, and socio-economic status) by examining the same population repeatedly over time. 

Because the daily emergency or outpatient visits typically follow a quasi-Poisson distribution, we used a generalized additive model (GAM) to examine their variations in response to air pollutants. We used a linear model to estimate the impacts of air pollution on medical expenditures. In both models, we applied the polynomial distributed lag models (DLM) to estimate the cumulative effects of air pollutants along lag days [10]. We decided on a maximum lag of three days (i.e., the present-day and previous three days) according to previous studies [1]. For each outcome, we incorporated the “cross-basis” function of a pollutant as an independent variable. Other covariates included: (1) a natural cubic smooth function of calendar time with seven degrees of freedom (df) per year to exclude potential annual trends and seasonality [11]; (2) an indicator variable for day of the week to account for the fluctuations of hospital visits within a week; (3) a binary dummy variable for holidays to account for the impacts of public holidays on hospital visits; (4) natural cubic smooth functions of daily temperature and humidity (6 df and 3 df for each) averaged from the present day to previous three days to control for their nonlinear and lagged confounding effects [1]. 

All statistical tests were two-sided, and values of *p* < 0.05 were considered statistically significant. Results were presented as the percentage increments in daily hospital visits, or increments (Chinese Yuan, CNY) in daily medical expenditure, in association with an interquartile range (IQR) increase of air pollutant concentrations. All analyses were performed using R software (version 3.3.1, R Foundation for Statistical Computing, http://cran.r-project.org/, Vienna, Austria) with the GAM fitted using the “mgcv” package and the DLM using the “dlnm” package.

## 3. Results

### 3.1. Descriptive Statistics

Table 1 summarizes the descriptive statistics of daily air pollutant concentrations and weather conditions in this study. During the study period from 1 January 2013 to 31 December 2015, we recorded a much higher level of PM_2.5_ (56 μg/m^3^) than that reported in developed countries. The annual-average concentrations of SO_2_, NO_2_, CO, and O_3_ were 19 μg/m^3^, 46 μg/m^3^, 821 μg/m^3^, and 100 μg/m^3^, respectively. There were positive and strong correlations among PM_2.5_, SO_2_, NO_2_, and CO (Pearson r: 0.75~0.89), but inverse and weak correlations with O_3_ (Pearson r: −0.22~−0.03). The annual average temperature and humidity were 17 °C and 68%, respectively, reflecting the subtropical climate in Shanghai. 

During the same period, on average, there were 230 emergency visits and 2100 outpatient visits due to respiratory diseases per day (Table 2). The daily average medical expenditures for respiratory outpatient visits were 73 thousand Chinese Yuan (CNY) and the daily expenditures for respiratory emergency visits were 488 thousand CNY (Table 2). For various categories of respiratory diseases, PRD accounted for the largest proportions (62–75%). Table 2 also shows an almost consistent seasonal pattern in daily visits and expenditures for the emergency- and outpatient-department due to total respiratory diseases, with a peak in winter and a trough in summer. 

### 3.2. Regression Results 

**Figure 1:**
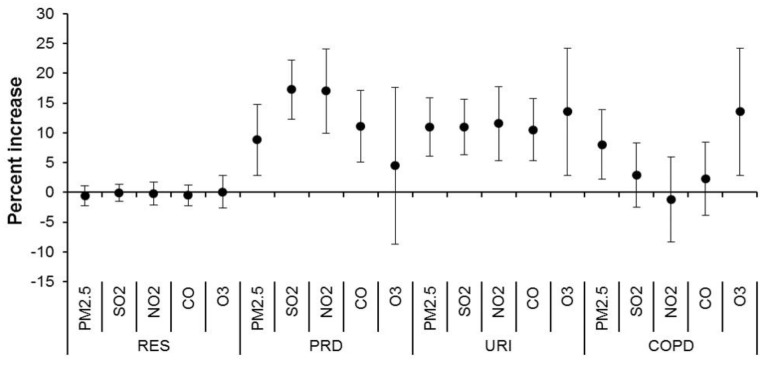
Cumulative percentage increase of daily emergency-room visits for respiratory diseases associated with an interquartile range increase in air pollutant concentrations over a lag of zero to three days. Abbreviations as shown in Table 1 and Table 2.

Figure 1 summarizes the estimated associations between air pollutants and emergency-room visits due to various respiratory diseases. Despite null associations between all air pollutants and total respiratory visits, we observed significant associations between all pollutants other than O_3_ and PRD visits, significant associations between all pollutants and URI visits, and significant associations between PM_2.5_ and O_3_ with COPD visits. There were non-significant associations of all pollutants with total respiratory diseases, asthma, and LRI. For an IQR increase in PM_2.5_, SO_2_, NO_2_, and CO, the cumulative increases for daily emergency visits due to PRD over lags of zero to three days were 8.81%, 17.26%, 17.02%, and 11.12%, respectively. The corresponding increases for daily emergency visits due to URI were 11.0–13.5%. PM_2.5_ and O_3_ were associated with 8.05% and 13.53% increases for daily emergency visits due to COPD.

**Figure 2:**
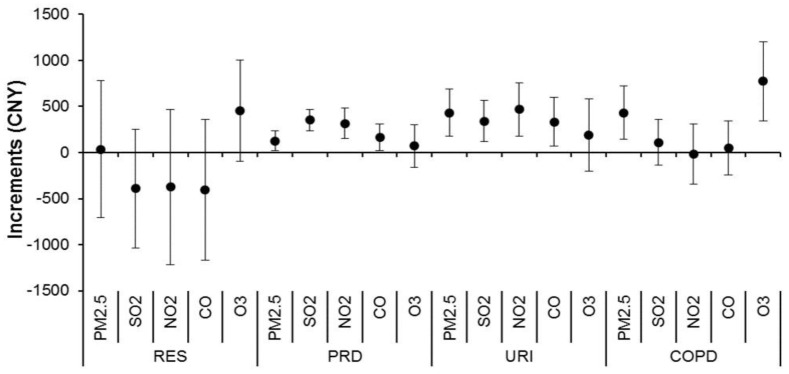
Cumulative increments of daily medical expenditure due to emergency-room visits for respiratory diseases associated with an interquartile range increase in air pollutant concentrations over a lag of zero to three days. Abbreviations as shown in Table 1 and Table 2.

Figure 2 shows the associations between air pollutants and medical expenditure due to daily respiratory emergency-room visits, which had similar patterns of associations with Figure 1. All pollutants other than O_3_ were significantly associated with emergency expenditure due to PRD visits with 128–352 CNY per IQR increments in air pollutants, accounting for 0.33–0.90% of daily mean expenditures. All pollutants other than O_3_ were significantly associated with emergency expenditure due to URI visits with 332–467 CNY per IQR increments in air pollutants, accounting for 9.32–13.08% of daily mean expenditures. PM_2.5_ and O_3_ were significantly associated with COPD visits with 432 and 774 CNY per respective IQR increments, accounting for 14.42–25.81% of daily mean expenditures (see the Graphical figure). There were non-significant associations of all pollutants with emergency expenditure due to total respiratory diseases, asthma, and LRI.

**Figure 3:**
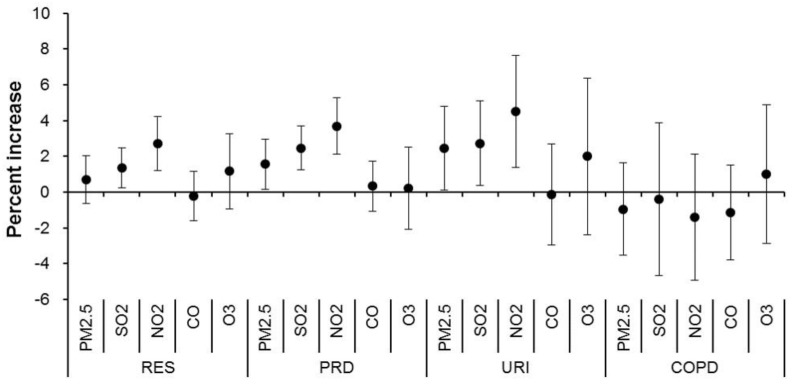
Cumulative percentage increase of daily outpatient visits for respiratory diseases associated with an interquartile range increase in air pollutant concentrations over a lag of zero to three days. Abbreviations as shown in Table 1 and Table 2.

Figure 3 illustrates the association between air pollutants and daily outpatient visits due to various respiratory diseases. Overall, we found appreciably lower effects of air pollutants on respiratory outpatient visits than on emergency visits. There were significant associations of SO_2_ and NO_2_ with total respiratory diseases, PRD, and URI. An IQR increase of SO_2_ and NO_2_ concentrations would lead to cumulative increments of 1.36–4.52% in total respiratory diseases, PRD, and URI over lags of zero to three days. PM_2.5_ was only marginally significantly associated with PRD and URI. O_3_ was not associated with any outcomes we examined. Air pollutants were not significantly associated with COPD, asthma, and LRI.

**Figure 4:**
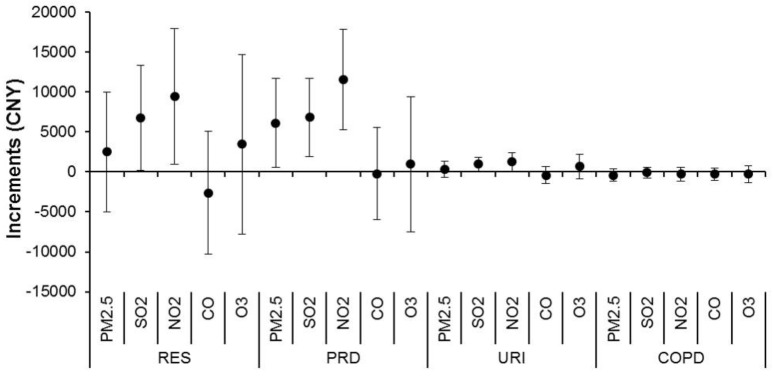
Cumulative increments of daily medical expenditure due to outpatient visits for respiratory diseases associated with an interquartile range increase in air pollutant concentrations over a lag of zero to three days. Abbreviations as shown in Table 1 and Table 2.

Figure 4 shows the associations between air pollutants and medical expenditure due to daily respiratory outpatient visits, which had similar patterns of associations with Figure 3. Generally, the expenditures for outpatient visits attributable to air pollution were considerably higher than those for emergency visits. An IQR increase in concentrations of SO_2_ and NO_2_ was significantly associated with increments of 6.14–11.58 thousand CNY for total RES and PRD. The corresponding proportions varied from 1.38 to 3.18% for daily mean expenditures. There were weaker or non-significant associations of air pollutants with outpatient expenditure due to COPD, asthma, and LRI.

## 4. Discussion

Outdoor air pollution represents a major and growing public health problem in China. This investigation found short-term exposure to criteria air pollutants was significantly associated with emergency-room visits due to PRD, URI, and COPD, and with outpatient visits due to PRD and URI in a large hospital in Shanghai, from 2013 to 2015. Accordingly, we estimated considerable medical expenditure for outpatient- or emergency-room visits associated with air pollution. However, we did not find significant effects of criteria air pollutants on asthma or lower respiratory infection. 

Epidemiological studies often adopt mortality to represent the health effects of air pollution. Compared with mortality, morbidity can be a more sensitive response to an exposure to air pollutants. Different morbidity outcomes typically including outpatient visits, emergency-room visits, and hospitalizations (or hospital admissions) have been evaluated in previous epidemiological studies [6,12]. In developed countries, the use of outpatient visits and hospitalizations in epidemiologic studies of acute effects of air pollution was somewhat problematic because hospital visits are usually scheduled by appointment and many patients visit doctors in local clinics rather than in hospitals. In contrary, in China, hospital visits are usually unscheduled, and are first-come first-served [12]. Noteworthy is that the number of hospitalizations might not vary appreciably from day to day because people in China always had a preference to admit in high-level hospitals, approximating to a saturation for inpatient beds. Therefore, outpatient- and emergency-room visits may reliably represent the true morbidity in China. The present study utilized the data from outpatient and emergency departments in a large hospital of Shanghai, offering a sufficient opportunity to evaluate the association between daily hospital visits and air pollution. 

The respiratory system is most vulnerable to the hazardous effects of air pollution, and respiratory diseases generally constitute major causes of hospital visits. A major advantage of this study was the inclusion of various causes of respiratory disease, which could avoid potential publication bias. It should be noted that we observed statistically significant associations for some, but not all, respiratory diseases. Compared with studies based on hospital admissions, fewer studies have directly evaluated cause-specific outpatient- and emergency-room visits as morbidity outcomes linked with air pollution exposure. Our results were comparable to some previous studies. For example, a time-series analysis in Beijing, China, estimated that every 10 μg/m^3^ increase in PM_2.5_ concentration was associated with an increase in emergency-room visits of 0.19% (95% CI: 0.04–0.35%) for URI and 1.46% (95% CI: 0.13–2.79%) for COPD [13]. A meta-analysis also supported almost consistent associations between air pollutants (particles, SO_2_ and NO_2_) on COPD-related morbidity [14]. Few studies have evaluated the effects of air pollutants on pediatric outpatient visits. Our results were consistent with a previous study conducted in Yichang, China, which estimated that each IQR increase in PM_2.5_, PM_10_, NO_2_, CO, and O_3_ concentrations was significantly associated with a 1.91%, 2.46%, 1.88%, 2.00%, and 1.91% increase of pediatric respiratory outpatient visits, respectively [15]. 

Recently, there have been many studies addressing the potential mechanisms whereby air pollution induces respiratory diseases. For example, air pollutants can impair respiratory epithelial cells and macrophages, possibly decreasing the host defenses to respiratory infection or increasing airway reactivity [16]. Also, a short-term exposure can lead to increased levels of fractionated exhaled nitric monoxide and hypo-methylation of its encoding gene, resulting in higher levels of airway inflammation [17]. Finally, we found generally larger effects of air pollutants on PRD, which may be attributable to the insufficient antioxidant defenses and weakened ability of scavenging exogenous toxicants in children [14]. 

Another important contribution of the present study was the monetization of the outpatient- and emergency-room visits caused by a short-term exposure to air pollution. Economic assessment on disease burden associated with air pollution has been focused on premature deaths [7,8]. To our knowledge, this study is the first to investigate the short-term effects of air pollution on medical expenditure for respiratory morbidity. Our study estimated significant increments (8.81–17.26%) for expenditures in emergency-room visits due to PRD, URI, and COPD, as well as smaller expenditures (1.38–3.18%) in outpatient visits for PRD and URI, for an IQR increase of air pollutants. Our study quantified the exposure-response associations between short-term exposure to air pollution and medical expenditures for various respiratory diseases. The estimated percent changes in medical expenditure may be helpful to monitor the disease burden and economic loss caused by air pollution and conduct further health impact assessment.

Our study has important public health implications. First, although the effects found in Shanghai are similar in magnitude, per amount of pollution, to those estimated in other parts of the world [18,19,20], the importance of these increased outpatient- and emergency-room visits is much greater than in developed countries because of the widespread high exposure levels of air pollution and the large size of the population in China. Second, our study evaluated the exposure-response associations between short-term exposure to all criteria air pollutants and outpatient- and emergency-room visits due to various respiratory diseases. The estimated percent changes may be helpful to monitor the disease burden caused by air pollution and conduct further health risk assessment in China. Third, our results emphasized an urgent demand to control air pollution and promote the use of personal protective equipment (e.g., respirators, air purifiers), which may be helpful to guide the susceptible subgroups (children, elders, and patients of chronic respiratory diseases) to take measures to protect their health in time.

Our study has several limitations. First, as in most previous time-series studies, we simply averaged the measurements across various fixed-site stations as the proxy for the population exposure level to air pollution in Shanghai. The simple averaging method could result in apparent exposure measurement errors given that monitoring measurements can differ from location to location and that they can also differ from personal exposure level [6,12]. The differences between these proxy values and the true exposures are an inherent and unavoidable type of measurement error, which was reported to underestimate the effects in time-series design [21,22]. Second, the data we collected were limited to one hospital in a city, reducing the generalizability of our results to other contexts; therefore, our findings (for example, the nonsignificant effects on asthma and lower respiratory infection) remain to be confirmed in more locations. Third, high correlations among air pollutants (except O_3_) in Shanghai limited our ability to separate the independent effect for each pollutant in the present study. Fourth, as in most epidemiological studies based on hospital visits, our results may be subject to the residual confounding in relation to differentiated access to medical care. Finally, as in previous epidemiological studies based on outpatient data, we were not able to exclude the bias associated with scheduled and regular outpatient visits.

## 5. Conclusions

In summary, the present study suggests that short-term exposure to outdoor air pollution may induce the occurrences or exacerbation of PRD, URI, and COPD, leading to considerable medical expenditures upon the patients. Our findings may contribute to key scientific information on air pollution-related health effects in China, thereby providing local decision-makers with informative priorities about public health measures with the largest health benefits. Our study highlights the demand for comprehensive information systems, including both air pollution monitoring/forecasting and health sectors (e.g., hospitals), to support such environmental accountability evaluation.

## Figures and Tables

**Table 1:** The descriptive statistics of daily air pollutant concentrations and weather conditions in this study.

	Mean	SD	Min	P25	P50	P75	Max
Pollutants (μg/m^3^)							
PM_2.5_	56	37	7	30	45	70	255
SO_2_	19	13	6	11	15	22	103
NO_2_	46	21	5	31	42	57	143
CO	821	311	364	606	735	951	2281
O_3_	100	44	11	69	96	124	266
Weather conditions							
Temperature (°C)	17	9	−1	10	18	24	35
Humidity (%)	72	13	31	63	73	81	98

Abbreviation: PM_2.5_, particulate matter with an aerodynamic diameter less than or equal to 2.5 μm; Sulfur dioxide, SO_2_; Nitrogen dioxide, NO_2_; Carbon monoxide, CO; Ozone, O_3_.

**Table 2:** The summary descriptive statistics (mean ± standard deviation) on daily visits and expenditures (×thousand, CNY) for the emergency- and outpatient-department due to respiratory diseases in this study.

Diseases	Emergency Department		Outpatient Department	
	Visits	Expenditures	Visits	Expenditures
Total RES	230 ± 85	73 ± 27	2100 ± 510	488 ± 153
PRD	14 ± 67	39 ± 18	1560 ± 340	364 ± 103
URI	17 ± 21	4 ± 5	170 ± 54	35 ± 13
COPD	5 ± 5	3 ± 3	50 ± 32	15 ± 12
Asthma	6 ± 3	2 ± 1	160 ± 74	55 ± 32
LRI	58 ± 18	25 ± 18	230 ± 85	56 ± 22
Total RES				
January	340 ± 99	113 ± 37	2520 ± 650	573 ± 197
February	280 ± 73	92 ± 24	1600 ± 510	347 ± 143
March	230 ± 46	75 ± 14	1830 ± 340	404 ± 99
April	260 ± 100	74 ± 22	1990 ± 310	447 ± 93
May	190 ± 60	58 ± 19	1990 ± 330	455 ± 100
June	170 ± 39	53 ± 13	2000 ± 290	458 ± 91
July	250 ± 88	77 ± 25	1960 ± 320	447 ± 102
August	240 ± 70	74 ± 22	1770 ± 320	403 ± 103
September	180 ± 46	57 ± 17	2050 ± 410	476 ± 125
October	180 ± 49	58 ± 14	2270 ± 430	541 ± 127
November	180 ± 39	58 ± 12	2440 ± 340	601 ± 109
December	270 ± 78	85 ± 21	2740 ± 410	686 ± 131

Abbreviations: RES, respiratory disease; PRD, pediatric respiratory diseases URI, Upper respiratory infection; COPD, chronic obstructive pulmonary disease; LRI, Lower respiratory infection; CNY, Chinese Yuan (also called CMB).
